# Research for the Optimal Flux-Cored Arc Welding Process of 9% Nickel Steel Using Multi Object Optimization with Solidification Crack Susceptibility

**DOI:** 10.3390/ma14071659

**Published:** 2021-03-28

**Authors:** Minho Park, Jisun Kim, Changmin Pyo, Joonsik Son, Jaewoong Kim

**Affiliations:** 1 Southwestern Branch Institute, Research Institute of Medium & Small Shipbuilding, Jeonnam 58457, Korea; mhpark@rims.re.kr (M.P.); jsson@rims.re.kr (J.S.); 2 Smart Manufacturing Process R&D Group, Korea Institute of Industrial Technology, Gwangju 61012, Korea; kimjisun@kitech.re.kr (J.K.); changmin@kitech.re.kr (C.P.)

**Keywords:** flux-cored arc welding, solidification crack susceptibility, 9% nickel steel (ASTM A553-1), multi object optimization, discriminant analysis

## Abstract

The environment of the global shipbuilding market is changing rapidly. Recently, the International Maritime Organization (IMO) has tightened regulations on sulfur oxide content standards for marine fuels and tightened sulfur oxide emission standards for the entire coastal region of China to consider the environment globally and use LNG as a fuel. There is a tendency for the number of vessels to operate to increase significantly. To use cryogenic LNG fuel, various pieces of equipment, such as storage tanks or valves, are required, and equipment using steel, which has excellent impact toughness in cryogenic environments, is required. Four steel types are specified in the IGG Code, and 9% Ni steel is mostly used for LNG fuel equipment. However, to secure safety at cryogenic temperatures, a systematic study investigating the causes of quality deterioration occurring in the 9% Ni steel welding process is required and a discrimination function capable of quality evaluation is urgent. Therefore, this study proposes a plan where the uniform quality of 9% Nickel steel is secured by reviewing the tendency of the solidification crack susceptibility among the quality problems of cryogenic steel to establish the criteria for quality deterioration and to develop a system capable of quality discrimination and defect avoidance.

## 1. Introduction

Recently, there has been a growing interest in preventing air pollution around the world and, as a result, the International Maritime Organization (IMO) is elevating regulations on marine sulfur oxides (SOX) and nitrogen oxides (NOX). In 2015, the emission concentration of sulfur oxides in the ECA (Emission Control Area) had already been strictly reduced from 1% to 0.1%, and the emission concentration of sulfur oxides in the high seas is expected to be reduced from 3.5% to 0.5% from 2020. Liquefied natural gas (LNG) is the most popular fuel that can smoothly comply with the regulations in such a situation and is currently evaluated as the only ship fuel that can meet the emission gas environmental regulations. Due to the characteristics of an LNG storage tank, which is a key facility in the LNG industry, there is no concept of overhaul once its operation is started under the current laws and standards. Therefore, it has the advantage of continuous operation for a lifetime. In Korea, LNG fuel is stably supplied according to KGS AC 115 (standards for facility, technology and inspection of LNG storage tank manufacturing) [1,2,3,4].

An LNG storage tank is mostly made of 9% Ni steel, and its strength level is classified as high-tensile steel and is used in applications with a temperature below −170 °C after QT (Quenching and Tempering) heat treatment. The 9% Ni steel is used for LNG tank production, as it has high impact toughness in cryogenic conditions, and its cost is inexpensive compared to its density. Due to these advantages, there are many production cases. The development of 9% Ni steel in Korea began in 1990, and now the steel is produced using the QLT (Quenching, Lamellaizing, and Tempering) method. A superior quality equal to or higher than those of advanced countries is secured, and 9% Ni steel is supplied to the Korean LNG tank industry [5,6].

In the 9% Ni welding process, the difficulty of the welding process is high, and the difference in the welding quality is large depending on the skill of a welding company. The advanced countries that have secured the 9% Ni steel welding technology keep their technologies thoroughly confidential. Therefore, research is necessary to secure advanced technology in the welding process and to derive uniform welding quality. Therefore, it is urgent to perform basic research to derive uniform, high-quality weldments by analyzing the phenomenon of welding quality degradation that may occur in the 9% Ni steel welding process and by investigating its causes. For these reasons, a former researcher, Bahador [7], also studied the negative effects of mechanical properties generated depending on the Ni content, ductility, and high crack sensitivity. Therefore, in this study, solidification crack susceptibility was defined by the weld quality standard and various analyses were applied to find the optimum weld parameters. For finding optimal parameters, solidification crack susceptibility was defined as the standard for welding quality. The lower the solidification crack susceptibility is, the better the welding quality is. In addition, the multipurpose optimization method is applied for searching the optimal welding condition.

Solidification cracks are a typical welding defect regardless of the welding of 9% Ni steel. This defect is due to the concentration phenomenon of solute elements in the residual melt and the shrinkage stress in the welding metal growth. This shrinkage generates residual stress and decreases the strength of the weldment due to impurities formed between the columnar grains. To resolve the quality deterioration of a weldment, optimization of the process by clarifying the correlation among the variables of the welding process and the weldment is needed.

Previous studies focused on the fiber laser welding process, but flux-cored arc welding (FCAW) for 9% Ni steel is more used in the field with many advantages. Therefore, this study aimed to analyze the characteristics of quality deterioration due to solidification cracks caused by the flux-cored arc welding process. A process variable optimization method was also suggested to avoid the strength degradation of a weldment.

Other related studies that were performed are summarized below. Kim [8] studied the weldability of 9% nickel steel for LNG storage tank used for carriers. Lee [9] researched the welding problem by the magnetization of 9% nickel steel. Kim [10] found that GMAW (gas metal arc welding) of 9% nickel steel improved the high temperature cracking susceptibility, but had a bad effect on the strength of the welded area. Saitoh [11] developed a high nickel-based steel with good toughness for cryogenic conditions. Kim [12] researched the design and weldability for LNG fueled tank with 9% nickel steel. Yun [13] searched the optimal welding process of fiber laser fillet welding with a gradient-based optimization method. Kim [14] applied the deep learning method to gas metal arc welding.

In the case of prior studies performed to date, there has been a review on the correlation between various variables applied to the welding process and mechanical properties for cryogenic steel, such as STS series or Ni alloy series, and also on the process problems and quality degradation that occur when they are used for LNG-related equipment. However, the studies on the quality of weldments related to cryogenic steels did not reflect the complex interaction effect and most of the studies focused on automation, high degree of welding, and high speed, etc., to compensate for the disadvantages of manual welding [15,16]. Of course, the research on process improvement for immediate use and productivity improvement in the field has an excellent academic impact. However, it is difficult to use for general purposes because there is no confirmation that excellent weld integrity and uniform quality are derived even when reflecting the process environment and characteristics of each different cryogenic steel welding site [17,18].

## 2. Experimental Works

**Figure 1:**
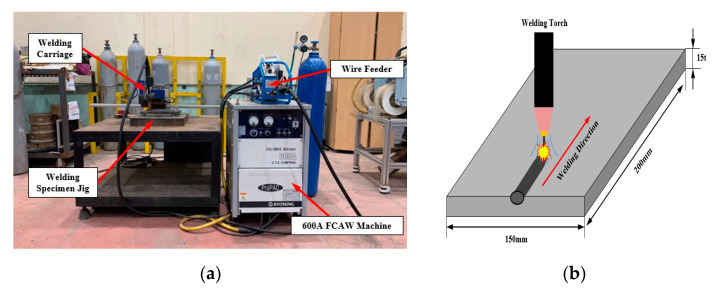
Equipment and process of flux-cored arc welding. (**a**) Experimental setup for flux-cored arc welding. (**b**) Flux-cored arc welding process.

In the experiment, a 600A class FCAW welding machine (ProPAC, HYOSUNG, Mapo-gu, Seoul, Korea) and torch, welding feeder, straight welding carriage, and a guide rail were configured. Ethyl alcohol (DUKSAN, Ansan-si, Gyeonggi-do, Korea) was used for cleaning the specimen, and sandpapers were applied for the same reason. Rust or oxide on the surface can cause welding defects. Figure 1 shows the experimental setup and the schematic diagram of the flux-cored arc welding process. The test piece used in the welding test was used in a size of 150 mm (W) × 200 mm(H) × 15 mm (H) of 9% Ni steel. Moreover, the chemical composition of 9% Ni steel and welding wire used in the test are shown in Table 1, and mechanical properties are shown in Table 2. Information on 9% Ni steel and welding wire was made using public data from manufacturers.

**Figure 2:**
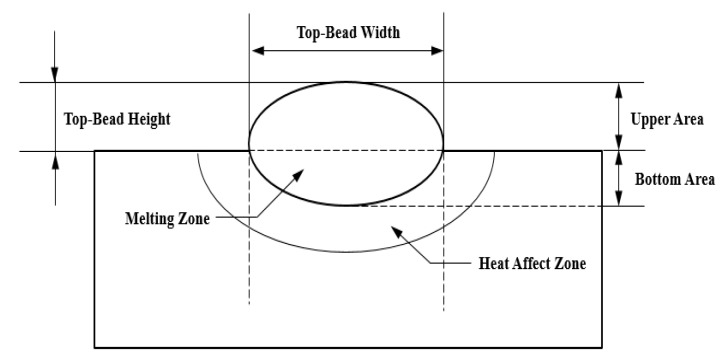
A schematic diagram of bead geometry.

As the input variables of the flux-cored arc welding applied in this experiment, the welding current, arc voltage, and welding speed were selected. These variables have a clear influence on the shape and weldability of a GMA weldment. The mechanical characteristics, such as the bead shape, hardness, impact amount, weldment component, and fracture surface, etc., were selected as output variables for weldability analysis. Figure 2 shows the bead shape of a weldment [19].

In this experiment, the full factorial design (FFD) was used, which can estimate all factor effects of the response of output variables according to the change in input variables and detect the high-order interaction effects. A full factorial design is a general K^n^ factorial method DOE with n number of factors and k level, and experiments are designed in a combination of levels between all factors. Therefore, even without repeated experiments, the number of K^n^ experiments should be performed. The factor experiment by the factor placement method has the advantage of being able to estimate all factor effects (main effects and interactions). The appropriate level and range of input variables (welding current, arc voltage, welding speed) were selected through preliminary experiments. Three different values of the welding current and two different values for the arc voltage were used, while for the welding speed, two different values were used, so the number of total experiments is 18 (3^2^ × 2 = 18). The levels of the input variables and the experimental conditions are shown in Table 3 and Table 4.

## 3. Results of Flux-Cored Arc Welding

### 3.1. Bead Geometry

The BOP (bead on plate) welding test was performed. To properly represent the cross-section of the test piece, a solution containing 90% ethanol and 10% nitric was used for etching the cross-section part and an optical microscope system was used for accurate bead shape measurement. Table 5 shows the weld cross-section and the bead shape taken with a 10× optical microscope. Top-bead geometry was measured according to Figure 2, and the measurement precision of the optical microscope used was 0.0001 mm.

### 3.2. Measurement of Hardness

A strength decrease in the welded area when the flux-cored arc welding was solidified can be checked with a hardness test and the impurities came upward due to the difference of density. As the upper part was vulnerable to the hardness because the impurities floated, in the upper part, the Vickers hardness test was applied.

**Figure 3:**
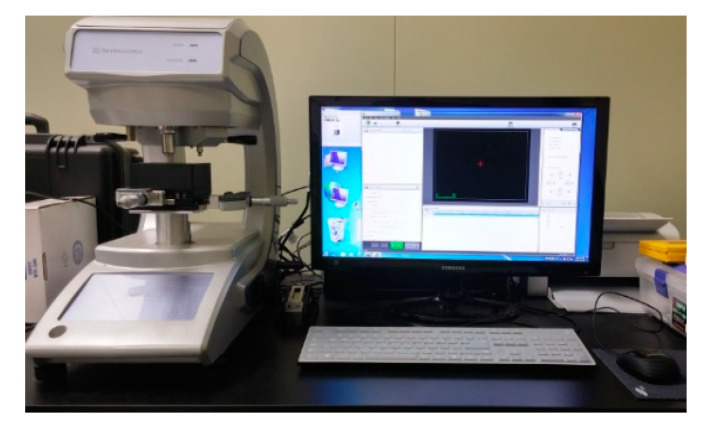
Vickers hardness tester.

**Figure 4:**
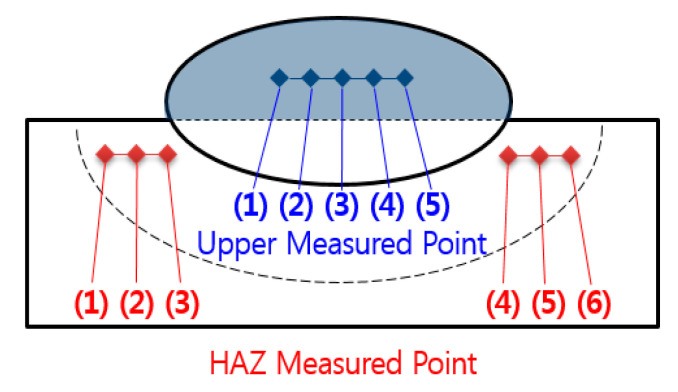
Measured points of the hardness test.

The load was 0.5 N and the intervals were 0.83 mm, which was for not affecting other measures. Figure 3 and Figure 4 show the tester and the points of the hardness test, and Table 6 shows the results of the upper part and the heat affected part. The upper hardness of a flux-cored arc weldment was between 250.1 and 262.6 Hv, which is considered to have sufficient weldability because the hardness is higher than the hardness 243 Hv, which is a standard of 9% Ni steel.

### 3.3. Measurement of Chemical Composition after Welding

**Figure 5:**
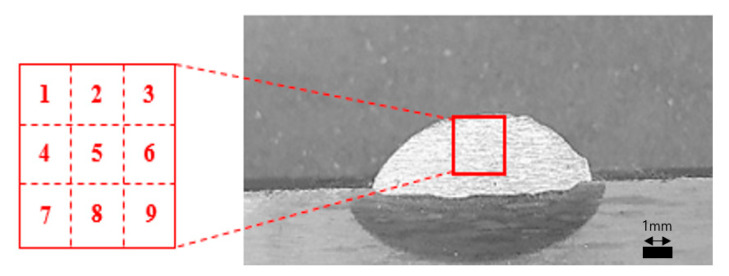
Measurement section of the welding bead.

**Figure 6:**
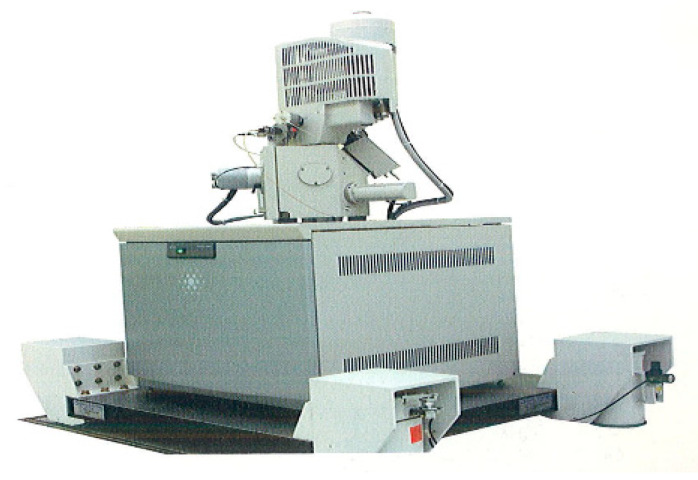
FE-ESEM tester for EDS analysis.

**Figure 7:**
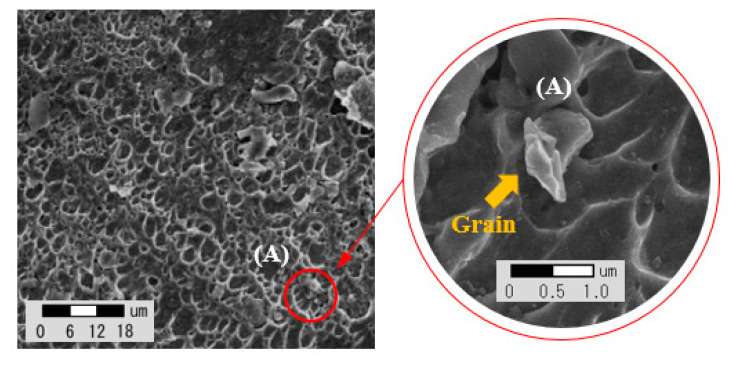
SEM image of the upper area after welding

To measure the impurities of Ti, Nb, Mo, and Si components that affect the crack susceptibility on the penetration and weld surface of the welding test piece, and to check the tendency of impurity grain boundaries that change according to the welding process variables, EDS was measured by dividing sections into nine points in Figure 5. In order to analyze the combination of various alloying elements and compositions, the influence of alloying elements on the microstructure and mechanical properties was analyzed. The FE-ESEM equipment shown in Figure 6 was used. The location of the component analysis was selected in consideration of the fact that it rises to the top due to the difference in density during the solidification process, and Figure 7 shows the grain boundaries of the upper impurities. Table 7 shows the average value of analysis for the four components, i.e., Ti, Nb, Mo, and Si.

## 4. Discriminant of Welding Quality

### 4.1. Solidification Crack Susceptibility

A nickel-based alloy has an austenite structure and tends to solidification crack. Thus, solving the solidification crack during the welding process of 9% Ni steel is a critical issue. The variables of the welding process are the main factors of the crack resistance to solidification crack, also cracks are more likely to occur with a higher welding current or operating ratio.

Nakao reviewed the solidification crack susceptibility of a nickel-based alloy in a melt welding, and formulated the correlation between crack susceptibility and impurity elements. That is solidification crack susceptibility index (P_SC_) described as Equation (1) [20].

P_SC =_ 69.2Ti + 27.3Nb + 9.7Mo + 300Si − 55.3 (1)

Because the flux-cored arc welding is a type of melt welding, the Psc was used to investigate the solidification crack susceptibility of 9% Ni steel. For the evaluation, the solidification crack susceptibility was calculated with Equation (1).

One of the purposes of this research was to confirm the phenomenon that the hardness of an upper weldment diminishes with the grain boundary relaxation when the crack susceptibility increases. It was also to define the criteria for crack susceptibility.

**Figure 8:**
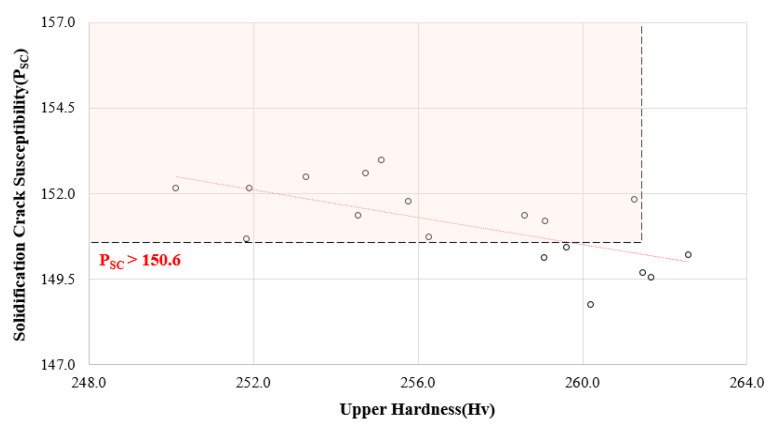
P_SC_ distributions depending on upper hardness in flux-cored arc welding.

Psc was between 148.7 and 153.0. Moreover, it was found that the hardness of an upper weldment was stable when Psc was 150.6 or less, as shown in Figure 8.

Psc can be used as an index of evaluation index, the score 150.6 is standard in this research. If it is a higher score, it means that there could be crack susceptibility for an upper weldment. This standardized score can be used to obtain data to establish the drop of grain boundary strength owing to crack susceptibility and can help prevent the micro-cracking with impurity grains in a 9% Ni steel weldment with the flux-cored arc welding (Table 8).

### 4.2. Discriminant Analysis

To discriminate the solidification crack susceptibility of the flux-cored are welding for 9% Ni steel, the discrimination model based on learning the data from experiments is developed, and it is used as an estimation model [21,22,23].

The solidification crack susceptibility discrimination system is based on the SVM (Support Vector Machine) technique to determine solidification cracking tendency by finding a hyperplane that maximizes a margin within two classes capable of linear separation based on Equation (2) in the Vapnik–Chervonenkis theory [24].

*w* · *x* + *b* = 0 (2)

The variables for learning in the discrimination model are the welding process (Welding Current, Arc Voltage, and Welding Speed), bead shape (Top-Bead Width, Top-Bead Height), hardness (upper part, heat-affected zone), and solidification crack susceptibility. One hundred sixty-two cases were used as input data with these nine variables. The Unstable Group, in terms of the solidification crack susceptibility, was defined as 1, and the other was defined as 0.

**Figure 9:**
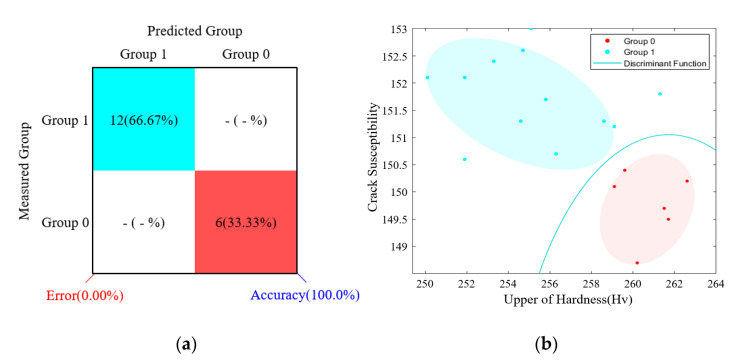
Performance of the discrimination model and discriminant function. (**a**) Performance of the discrimination model and (**b**) discriminant function.

Table 9 shows the learning data, and Table 10 shows the difference between the measured result and the predicted result. Figure 9 shows the performance of the discrimination model.

## 5. Optimization of the Flux-Cored Arc Welding Process

### 5.1. Mathematical Model for Optimization

To optimize the flux-cored arc welding process, the interaction formula among the input variables and objective function value was defined. The response surface method is known to be proper to the multi-input variables cases, it is applied to this research as in the previous research, which is related to fiber laser welding [25]. The method of calculating the estimated values of $\beta_{0}$ and $\beta_{1}$ that minimizes the sum of squares of the residual e, which is the deviation between the observed value Y and the estimated value of ${\hat{Y_{i}}}_{\;}$, is called the method of least squares. That is, if the sum of squares of the residuals is S as follows, if S is partially differentiated and summarized, the least-squares estimate, $\beta_{\;}$, is obtained.

The functional relationship between the input variable $x_{1},\;x_{2},x_{3},\;\cdots x_{k}$ and the output variable y is expressed in Equation (3). This research also used the second-order regression model, as shown in Equation (4).

(3) $$Y_{i}\;=\;f{\left(x_{1},x_{2},x_{3}\right)}_{\;}$$

(4) $$Y_{i}\;=\;\beta_{0}+\overset{k}{\underset{i\;=\;1}{\sum }}\beta_{i}k_{i}+\overset{k}{\underset{i\leq j}{\sum }}\beta_{ij}x_{i}x_{j}+\epsilon$$

By the least squares method, Equation (4) is replaced by Equation (5).

(5) $$\hat{Y_{i}}\;=\;\hat{\beta_{i}}+\overset{k}{\underset{i\;=\;1}{\sum }}\hat{\beta_{i}}k_{i}+\overset{k}{\underset{i\leq j}{\sum }}\hat{\beta_{ij}}x_{i}x_{j}+c$$

when the number of input variables is 3, k is 3 and Equation (5) changes to Equation (6).

(6) $$\begin{array}{c} \hat{Y_{i}}\;=\;\hat{\beta_{0}}+\hat{\beta_{1}}x_{1}+\hat{\beta_{2}}x_{2}+\hat{\beta_{3}}x_{3}+\hat{\beta_{11}}x_{1}^{2}+\hat{\beta_{22}}x_{2}^{2}+\hat{\beta_{33}}x_{3}^{2}\\ +\hat{\beta_{12}}x_{1}x_{2}+\hat{\beta_{13}}x_{1}x_{3}+\hat{\beta_{23}}x_{2}x_{3} \end{array}$$

$\hat{Y_{i}}$ is the output variable such as bead dimensions, hardness, Psc, $x_{i}$ are input variables such as welding process variables, $\hat{\beta_{0}},\;\hat{\beta_{i}},\;\hat{\beta_{ij}}$ are the least square estimates of $\beta_{0},\;\beta_{i},\;\beta_{ij}$, and ϵ is an error term. To complete Equation (6), relevant data should be needed with many experiments.

For efficient data acquisition, a complete factor design that is proper to the second-order regression model was applied. The coefficient of each term was obtained with Minitab. With the above theories, the prediction model of bead shape (Top-Bead Width, Top-Bead Height), hardness (upper part, HAZ), and solidification crack susceptibility were expressed as Equation (7) to Equation (11).

(7) $$\begin{array}{c} W\;=\;4.0366-0.1264C+0.0546V-8.5917S+0.0004C^{2}-0.0115V^{2}\\ +0.0058CV+0.1333CS-0.4250VS \end{array}$$

(8) $$\begin{array}{c} H\;=\;2.1259-0.0802C+0.3100V+8.3833S+0.0004C^{2}-0.0021V^{2}\\ -0.0007CV-0.0283CS-0.1500VS \end{array}$$

(9) $$\begin{array}{c} H_{U}\;=\;1386.8-1.6263C-232.99W+2017.2H+0.0186C^{2}-9.6578W^{2}\\ -621.11H^{2}-0.2596CW-0.3473CH+161.33WH \end{array}$$

(10) $$\begin{array}{c} H_{H}\;=\;-1727.9+13.931C-215.88W+1385.1H-0.0369C^{2}-7.8645W^{2}\\ -328.98H^{2}+0.6200CW-2.7470CH+95.326WH \end{array}$$

(11) $$\begin{array}{c} P_{\mathrm{SC}}\;=\;-402.04+257.89H+32.887H_{U}-23.668H_{H}+12.489H^{2}-0.0737H_{U}^{2}\\ +0.0422H_{H}^{2}+1.2576HH_{U}-1.9294HH_{H}+0.0029H_{U}H_{H} \end{array}$$

**Figure 10:**
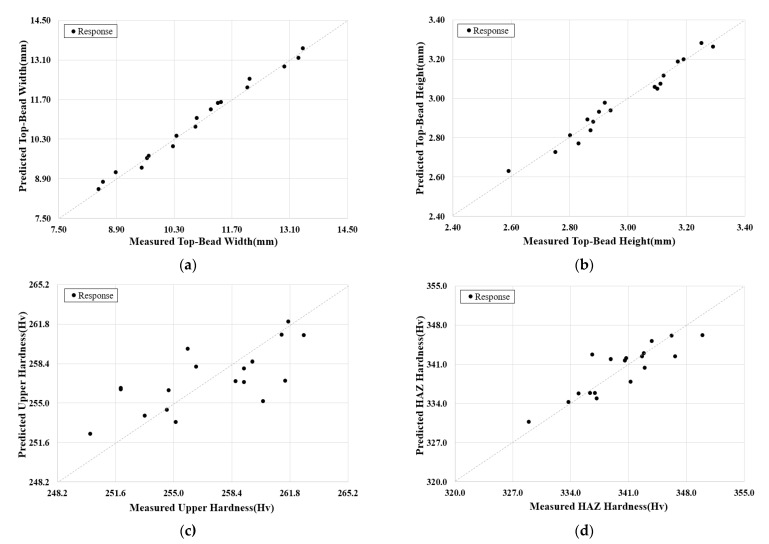

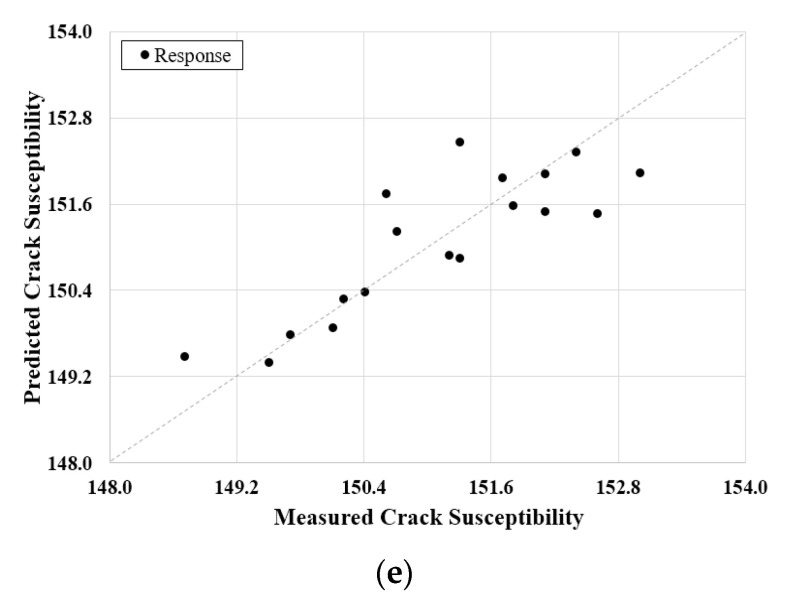
Comparison between measured and predicted welding parameters: (**a**) Top-Bead width, (**b**) Top-Bead height, (**c**) Hardness of upper part, (**d**) Hardness of HAZ, and (**e**) Crack susceptibility.

To confirm the consistency of the prediction models, Figure 10 shows the error range by comparing the average value of the measured welding factors and the predicted welding factors. The prediction model error range is generally reliable, which is shown in Table 11.

Besides, the result of variance analysis of the prediction model confirmed 98.9% in top-bead width and 73.0% in the upper hardness of a weldment. That means the interaction of the input variables is also considered.

### 5.2. Optimization of the Welding Process

In this research, the multi-objective optimization (MOO) algorithm is applied, which is known to be proper to solve the optimization problems with multiple purposes [26,27,28]. As the previous research related to the optimization of the fiber laser welding process used that algorithm for optimization and described that technique [25], this article omitted the details. In short, that technique imitated the evolution process in the ecosystem, the weighted sum method was used for solving the multi-objective problem.

**Figure 11:**
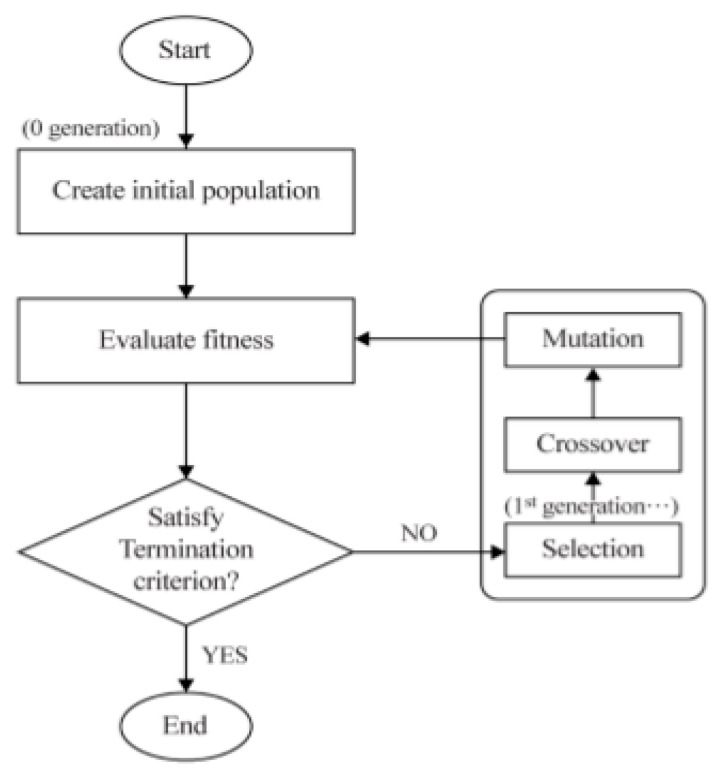
A flowchart of the Multi-Objective Optimization (MOO). Reprinted with permission from ref. [25].

In Figure 11 MOO algorithm was described, and MATLAB was used. To optimize the welding process variables, the 162 data points described in Table 9 were used. The variables and levels for the MOO algorithm are shown in Table 12.

The range of flux-cored arc welding process variables was selected from the minimum (150 A, 21 V, 0.3 m/min) to the maximum (170 A, 25 V, 0.4 m/min). The aim was to analyze a multi-purpose optimization problem, which considers the solidification crack susceptibility as a standard to access the quality deterioration characteristics after welding. The objective function is mathematical modeling of system characteristics, and its constraints represent the conditions that the system variables can have. Therefore, Equation (12), Equation (13), and Equation (14), respectively, show the objective function $f{\left(x\right)}_{\;}$of an arbitrary system having $x_{\;}$as a variable and the constraints and ranges required to optimize the function [29].

(12) Optimize f(C, V, S)

(13) g(C, V, S)

(14) $$P_{SC}<150.6$$

The cases where the solidification crack susceptibility occurred were selected for verifying the MOO algorithm. The solidification crack susceptibility occurred in Tests 2, 6, and 14, the improvement of the welding process through the optimization algorithm was checked. Table 13 shows the improvement with changing the variables, such as C, V, and S, also shows that Psc is lower than 150.6, respectively.

**Figure 12:**
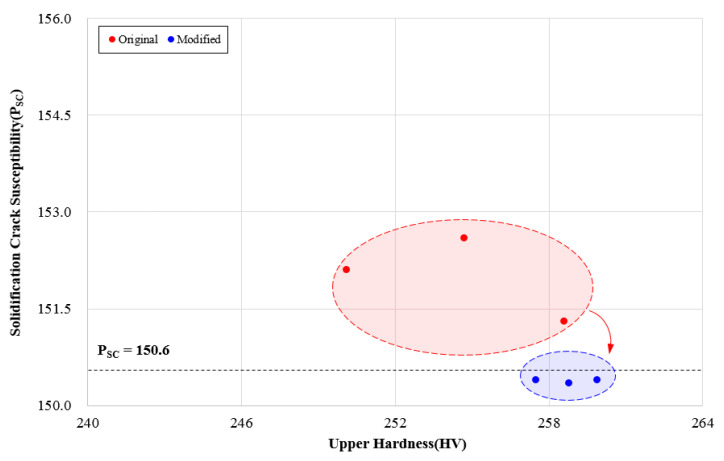
Crack susceptibility distributions using modified input parameters.

Figure 12 shows the attempt to confirm the solidification crack susceptibility by applying the improved input variables. It was confirmed that all points selected in the flux-cored arc welding process satisfy the solidification crack susceptibility limit condition of 150.6 or less. Moreover, the quality deterioration characteristics that appeared in the existing process variables are improved by the modified variables.

## 6. Conclusions

The following objectives were attempted in this study: To optimize the FCAW process for 9% Ni steel used in the cryogenic condition, to establish the criteria for the solidification crack susceptibility in the welding process, to develop learning in the discrimination function, and to optimize the variables that cause solidification crack susceptibility. Thus, the following results were obtained.

(1) Appropriate weldability was checked by measuring the bead shape, mechanical strength, and chemical composition. The solidification crack susceptibility was suggested as a standard of welding quality. When that index is 150.6 or more, it is difficult to secure a stable upper hardness.

(2) To determine the solidification crack, the SVM technique was used to check whether it can accurately identify a group where quality deterioration occurs. The accuracy of the prediction model was checked and verified.

(3) A prediction model based on the response surface method was suggested, it is applied to the optimization method. Multi-objective optimization algorithm was also used and verified.

## Figures and Tables

**Table 1:** Chemical composition of 9% Ni steel. (wt.%)

Material	C	Si	Mn	S	P	Ni	Fe
9% Ni	0.05	0.67	0.004	0.003	0.25	9.02	Bal.
Welding Wire	0.02	0.02	0.1	0.001	0.001	69.8	5.6

**Table 2:** Mechanical properties of 9% Ni steel.

Material	Yield Strength (MPa)	Tensile Strength (MPa)	Elongation (%)	Hardness (HV)
9% Ni	651.6	701.1	26.6	243
Welding Wire	460	730	47	230

**Table 3:** Flux-cored arc welding variables and their levels.

Parameter	Symbol	−1	0	1
Welding Current (A)	C	150	160	170
Arc Voltage (V)	V	21	23	25
Welding Speed (meter/minute)	S	0.3	−	0.4
Fixed Parameter	Welding Wire: ∅1.2 Flux Wire			
	Contact Tip Work Distance: 15 mm			
	Shielding Gas Flow Rate: 18 L/min			

**Table 4:** Experimental conditions of flux-cored arc welding.

Test No.	C	V	S	Test No.	C	V	S
1	150	21	0.3	10	150	21	0.4
2	150	23	0.3	11	150	23	0.4
3	150	25	0.3	12	150	25	0.4
4	160	21	0.3	13	160	21	0.4
5	160	23	0.3	14	160	23	0.4
6	160	25	0.3	15	160	25	0.4
7	170	21	0.3	16	170	21	0.4
8	170	23	0.3	17	170	23	0.4
9	170	25	0.3	18	170	25	0.4

**Table 5:** Dimensions of bead of flux-cored arc welding experiment.

Test No.	Top-Bead Width (mm)				Top-Bead Height (mm)				Bead Geometry
	1st	2nd	3rd	Average	1st	2nd	3rd	Average	
1	8.46	8.47	8.46	8.46	2.58	2.61	2.59	2.59	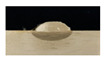
2	8.91	8.85	8.88	8.88	2.83	2.82	2.84	2.83	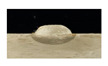
3	9.64	9.63	9.63	9.63	2.85	2.83	2.88	2.86	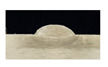
4	10.25	10.25	10.28	10.26	2.82	2.80	2.77	2.80	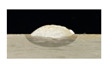
5	10.82	10.81	10.81	10.81	2.94	2.96	2.92	2.94	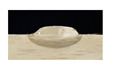
6	11.19	11.18	11.18	11.18	3.09	3.10	3.10	3.10	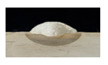
7	11.43	11.44	11.43	11.43	3.12	3.11	3.09	3.11	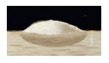
8	12.09	12.13	12.14	12.12	3.17	3.14	3.19	3.17	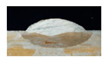
9	13.28	13.31	13.30	13.30	3.26	3.26	3.24	3.25	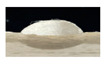
10	8.57	8.58	8.56	8.57	2.75	2.76	2.74	2.75	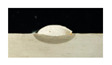
11	9.56	9.53	9.45	9.51	2.85	2.86	2.90	2.87	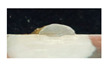
12	9.70	9.71	9.64	9.68	2.89	2.91	2.91	2.90	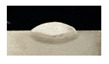
13	10.32	10.35	10.38	10.35	2.88	2.89	2.87	2.88	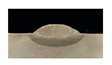
14	10.88	10.84	10.81	10.84	2.89	2.92	2.94	2.92	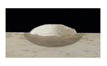
15	11.38	11.35	11.32	11.35	3.09	3.09	3.08	3.09	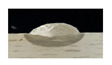
16	12.06	12.05	12.09	12.07	3.13	3.12	3.11	3.12	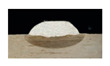
17	12.97	12.96	12.94	12.96	3.21	3.19	3.17	3.19	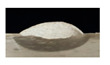
18	13.42	13.40	13.41	13.41	3.27	3.28	3.31	3.29	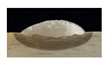

**Table 6:** Hardness test results.

Test No.	Upper Part (Hv)						Test No.	Heat Affected Zone (HAZ) (Hv)						
	1st	2nd	3rd	4th	5th	Avg.		1st	2nd	3rd	4th	5th	6th	Avg.
1	256.1	254.2	252.9	253.8	249.5	253.3	1	374.1	373.4	373.1	373.2	373.6	373.7	373.5
2	251.6	247.3	253.2	250.8	247.8	250.1	2	379.6	380.8	379.8	380.0	380.0	379.6	380.0
3	250.5	250.9	253.0	252.8	252.0	251.9	3	379.8	379.9	380.4	381.0	379.8	380.6	380.3
4	252.9	253.9	255.8	258.1	254.8	255.1	4	384.1	384.1	384.4	384.0	384.7	384.3	384.3
5	255.6	260.0	261.2	262.0	259.2	259.6	5	382.4	382.8	383.4	382.8	382.4	382.7	382.7
6	253.9	253.9	255.0	253.0	257.9	254.7	6	376.1	376.5	376.5	376.3	377.2	376.8	376.6
7	264.6	260.3	261.4	255.7	266.4	261.7	7	386.3	386.3	386.8	386.0	386.6	385.9	386.3
8	264.8	262.4	260.6	259.5	265.6	262.6	8	385.4	385.4	385.0	385.6	385.0	385.8	385.4
9	260.6	261.0	262.4	259.3	264.0	261.5	9	377.5	377.4	376.3	378.5	377.6	378.5	377.7
10	258.3	254.6	260.9	259.0	262.7	259.1	10	372.4	371.9	373.0	371.5	371.8	371.5	372.0
11	264.0	257.6	260.1	262.9	256.4	260.2	11	371.4	371.8	370.8	371.3	371.3	371.2	371.3
12	256.2	253.3	253.9	254.8	254.7	254.6	12	372.6	371.8	371.7	372.3	371.2	372.7	372.1
13	256.6	256.2	254.1	253.8	258.3	255.8	13	373.0	373.2	372.6	373.0	373.6	372.8	373.1
14	256.8	260.9	259.6	257.6	258.2	258.6	14	373.6	373.4	373.5	374.0	372.6	373.1	373.4
15	260.5	259.6	257.6	260.9	256.9	259.1	15	371.9	372.8	371.6	371.8	372.5	372.0	372.1
16	258.7	263.7	267.6	257.2	259.1	261.3	16	375.7	375.5	376.0	375.3	375.6	374.9	375.5
17	249.2	255.5	249.9	254.6	250.4	251.9	17	381.4	381.2	381.9	373.0	373.8	372.4	377.3
18	258.6	255.3	254.2	259.2	254.1	256.3	18	375.9	376.3	376.2	374.9	374.9	375.8	375.7

**Table 7:** Chemical properties after welding. (wt.%).

Test No.	Ti	Nb	Mo	Si
1	0.0206	0.1025	0.1536	0.6735
2	0.0207	0.1048	0.1535	0.6722
3	0.0207	0.1063	0.1525	0.6671
4	0.0205	0.1043	0.1538	0.6750
5	0.0204	0.1050	0.1526	0.6665
6	0.0205	0.1045	0.1533	0.6737
7	0.0206	0.1034	0.1535	0.6637
8	0.0205	0.1054	0.1527	0.6657
9	0.0205	0.1046	0.1537	0.6640
10	0.0206	0.1060	0.1536	0.6653
11	0.0206	0.1055	0.1525	0.6608
12	0.0205	0.1053	0.1523	0.6696
13	0.0205	0.1064	0.1531	0.6707
14	0.0204	0.1034	0.1530	0.6698
15	0.0205	0.1049	0.1529	0.6690
16	0.0205	0.1041	0.1525	0.6712
17	0.0204	0.1049	0.1528	0.6723
18	0.0204	0.1054	0.1533	0.6675

**Table 8:** Solidification crack susceptibility data.

Test No.	Upper Hardness (Hv)	P_SC_ Value	Crack Susceptibility	Test No.	Upper Hardness (Hv)	P_SC_ Value	Crack Susceptibility
1	253.3	152.4	Unstable	10	259.1	150.1	Stable
2	250.1	152.1	Unstable	11	260.2	148.7	Stable
3	251.9	150.6	Unstable	12	254.6	151.3	Unstable
4	255.1	153.0	Unstable	13	255.8	151.7	Unstable
5	259.6	150.4	Stable	14	258.6	151.3	Unstable
6	254.7	152.6	Unstable	15	259.1	151.2	Unstable
7	261.7	149.5	Stable	16	261.3	151.8	Unstable
8	262.6	150.2	Stable	17	251.9	152.1	Unstable
9	261.5	149.7	Stable	18	256.3	150.7	Unstable

**Table 9:** Learning data for discrimination model.

Test No.	*C*	*V*	*S*	*W*	*H*	*H*_U_	*H*_H_	*P*_SC_	Group
1	150.0	21.0	0.3	8.46	2.59	253.3	373.5	152.4	Unstable
2	150.0	23.0	0.3	8.88	2.83	250.1	380.0	152.1	Unstable
3	150.0	25.0	0.3	9.63	2.86	251.9	380.3	150.6	Unstable
4	160.0	21.0	0.3	10.26	2.80	255.1	384.3	153.0	Unstable
5	160.0	23.0	0.3	10.81	2.94	259.6	382.7	150.4	Stable
6	160.0	25.0	0.3	11.18	3.10	254.7	376.6	152.6	Unstable
7	170.0	21.0	0.3	11.43	3.11	261.7	386.3	149.5	Stable
8	170.0	23.0	0.3	12.12	3.17	262.6	385.4	150.2	Stable
9	170.0	25.0	0.3	13.30	3.25	261.5	377.7	149.7	Stable
10	150.0	21.0	0.4	8.57	2.75	259.1	372.0	150.1	Stable
11	150.0	23.0	0.4	9.51	2.87	260.2	371.3	148.7	Stable
12	150.0	25.0	0.4	9.68	2.90	254.6	372.1	151.3	Unstable
13	160.0	21.0	0.4	10.35	2.88	255.8	373.1	151.7	Unstable
14	160.0	23.0	0.4	10.84	2.92	258.6	373.4	151.3	Unstable
15	160.0	25.0	0.4	11.35	3.09	259.1	372.1	151.2	Unstable
16	170.0	21.0	0.4	12.07	3.12	261.3	375.5	151.8	Unstable
17	170.0	23.0	0.4	12.96	3.19	251.9	377.3	152.1	Unstable
18	170.0	25.0	0.4	13.41	3.29	256.3	375.7	150.7	Unstable

*C:* Welding Current (A);·*V*: Arc Voltage (V);·*S*: Welding Speed (m/min); *W*: Top-Bead Width (mm); *H*: Top-Bead Height (mm); *H*_U_: Upper Hardness (Hv); *H*_H_: HAZ Hardness (Hv); ·*P*_SC_: Solidification Crack Susceptibility.

**Table 10:** Comparison of the measured results and predicted results with the Support Vector Machine (SVM) technique.

Test No.	Measured	Predicted	Test No.	Measured	Predicted
1	1	1.00	10	0	0.00
2	1	1.00	11	0	0.00
3	1	1.00	12	1	1.00
4	1	1.00	13	1	1.00
5	0	0.00	14	1	1.00
6	1	1.00	15	1	1.00
7	0	0.00	16	1	1.00
8	0	0.00	17	1	1.00
9	0	0.00	18	1	1.00

**Table 11:** Results of verification of the predicted model.

Design Parameter	SE (Standard Error)	R^2^ (Coefficient of Determination, %)
*W*	0.221	98.9
*H*	0.046	96.9
*H*_U_	0.098	73.0
*H*_H_	3.697	76.2
*P*_SC_	3.735	75.4

**Table 12:** Parameters and their values for MOO.

Parameters		Values
Range of Local Parameters	*W* (Top-Bead Width)	(−5 ≤ Input ≤ +5) A
	*H* (Top-Bead Height)	(−1 ≤ Input ≤ +1) V
	*S* (Welding Speed)	(−0.05 ≤ Input ≤ +0.05) m/min
Range of Constraints	*P*_SC_ (Crack Susceptibility)	*P*_SC_ ≤ 150.6
Fitness Factor	Population Size	50, 60, 70, 80, 90, 100
Solver		Constrained nonlinear minimization
Algorithm		Trust region reflective algorithm
Derivatives		Gradient supplied

**Table 13:** Improvement through optimization.

Test No.	Original			Modified			Welding Factors					Group
	*C*	*V*	*S*	*C*	*V*	*S*	*W*	*H*	*H*_U_	*H*_H_	*P*_SC_	
2	150.0	23.0	0.3	151.1	20.2	0.35	8.6	2.6	257.5	334.0	149.2	Stable
6	160.0	25.0	0.3	164.9	24.0	0.25	11.8	3.1	259.9	344.7	150.4	Stable
14	160.0	23.0	0.4	155.0	22.0	0.39	9.9	2.9	258.8	339.4	150.1	Stable

## Data Availability

The data presented in this study are available on request from the corresponding author.
